# A voxel-wise assessment of growth differences in infants developing autism spectrum disorder

**DOI:** 10.1016/j.nicl.2020.102551

**Published:** 2020-12-29

**Authors:** A. Cárdenas-de-la-Parra, J.D. Lewis, V.S. Fonov, K.N. Botteron, R.C. McKinstry, G. Gerig, J.R. Pruett, S.R. Dager, J.T. Elison, M.A. Styner, A.C. Evans, J. Piven, D.L Collins, for the IBIS Network

**Affiliations:** a Montreal Neurological Institute, McGill University, Montreal, Quebec H3A 0G4, Canada; b Mallinckrodt Institute of Radiology, Washington University, St. Louis, MO 63110, USA; c Tandon School of Engineering, New York University, New York, New York 10003, USA; d Department of Psychiatry, Washington University School of Medicine, St. Louis, MO 63110, USA; e Department of Radiology, University of Washington, Seattle, WA 98105, USA; f Institute of Child Development, University of Minnesota, Minneapolis, MN 55455, USA; g Department of Psychiatry, University of North Carolina, Chapel Hill, NC 27599, USA

**Keywords:** Autism spectrum disorder, Neurodevelopmental disorders, Tensor based morphometry, Longitudinal neuroimaging

## Abstract

Autism Spectrum Disorder (ASD) is a phenotypically and etiologically heterogeneous developmental disorder typically diagnosed around 4 years of age. The development of biomarkers to help in earlier, presymptomatic diagnosis could facilitate earlier identification and therefore earlier intervention and may lead to better outcomes, as well as providing information to help better understand the underlying mechanisms of ASD. In this study, magnetic resonance imaging (MRI) scans of infants at high familial risk, from the Infant Brain Imaging Study (IBIS), at 6, 12 and 24 months of age were included in a morphological analysis, fitting a mixed-effects model to Tensor Based Morphometry (TBM) results to obtain voxel-wise growth trajectories. Subjects were grouped by familial risk and clinical diagnosis at 2 years of age. Several regions, including the posterior cingulate gyrus, the cingulum, the fusiform gyrus, and the precentral gyrus, showed a significant effect for the interaction of group and age associated with ASD, either as an increased or a decreased growth rate of the cerebrum. In general, our results showed increased growth rate within white matter with decreased growth rate found mostly in grey matter. Overall, the regions showing increased growth rate were larger and more numerous than those with decreased growth rate. These results detail, at the voxel level, differences in brain growth trajectories in ASD during the first years of life, previously reported in terms of overall brain volume and surface area.

## Highlights

• Pediatric neuroimaging study of Autism Spectrum Disorder.
• Longitudinal Tensor Based Morphometry of the presymptomatic period of ASD.
• Differences in voxelwise growth trajectories of children with ASD.
• Regions with differences have been implicated in the core symptoms of ASD.

## 1 Introduction

Autism Spectrum Disorder (ASD) is a complex neurodevelopmental disorder that is typically diagnosed around 4 years of age (Maenner et al., 2020). However, there continues to be no clear understanding of its underlying causes (Buxbaum and Hof, 2013). ASD is characterized by impaired social communication and interaction, as well as repetitive patterns of behavior, and restricted interests and activities (American Psychiatric Association, 2013) and a characteristic course. Inherent to ASD is the presence of wide phenotypic heterogeneity, which may be one of the factors complicating early identification. A central effort in the field is to improve identification prior to manifestation of behavioral symptoms to facilitate entry into early intervention services (Zwaigenbaum et al., 2015). Indeed, early behavioural intervention in children with ASD has been shown to improve their acquisition of language and play skills (Ben-Itzchak and Zachor, 2007, Schreibman et al., 2015, Shire et al., 2017), their overall social behaviour (Dawson et al., 2012, Dawson et al., 2010), and overall intellectual ability (Estes et al., 2015a). Thus, our goal is to characterize morphological differences of the brain across a period of development that includes a presymptomatic period and the emergence and consolidation of the diagnostic profile. Identifying putative biomarkers that distinguish high-risk infants who later receive a diagnosis may augment early identification efforts. Further, identifying brain-based biomarkers has the potential to inform models of pathogenesis and may elucidate novel targets for early intervention.

Several studies established replicable morphological differences in ASD. Some of the most consistent and prominent findings include larger head circumferences (Bailey et al., 1993, Davidovitch et al., 1996, Elder et al., 2008, Hazlett et al., 2005, Lainhart et al., 1997, Stevenson et al., 1997, Woodhouse et al., 1996) and brain volumes (Carper et al., 2002, Hazlett et al., 2005, Piven et al., 1996, Piven et al., 1995, Piven et al., 1992, Redcay and Courchesne, 2005, Sparks et al., 2002).

A number of regions have been found to have differences in ASD, including the corpus callosum (Frazier et al., 2012, Hardan et al., 2009, 2000; Manes et al., 1999, Piven et al., 1997, Wolff et al., 2015), the amygdala and hippocampus (Aylward et al., 1999, Barnea-Goraly et al., 2014, Dager et al., 2007, Kim et al., 2010, Schumann et al., 2009, Sparks et al., 2002), and the cerebellum (Bauman and Kemper, 1985, Carper and Courchesne, 2000, D’Mello et al., 2015, Fatemi et al., 2012, Piven et al., 1992, Stoodley, 2014, Wang et al., 2014). The nature of these differences, however, is sometimes conflicting across different studies. For example, the corpus callosum has been variously reported as reduced in cross-sectional area or volume in the anterior region only (Hardan et al., 2000), posterior region only (Piven et al., 1997) or in its whole volume (Boger-Megiddo et al., 2006, Hardan et al., 2009).

These apparently conflicting results may be explained in part by cohort age differences between studies, highlighting the importance of longitudinal studies starting at a very young age. In the case of children between 6 and 24 months of age, a recent study found the corpus callosum to be enlarged at 6 months with no significant difference by 24 months of age (Wolff et al., 2015), in contrast with most studies reporting decreased corpus callosum size in young children and older subjects with ASD (Frazier et al., 2012, Hardan et al., 2009). In addition, some studies have consistently reported an early increase in brain size (Courchesne et al., 2011, Redcay and Courchesne, 2005) and growth rate during childhood (Hazlett et al., 2011, Hazlett et al., 2012b, Piven et al., 1996), however, there are few studies that have focused on subjects younger than 24 months of age, where early diagnosis may be difficult but of particular clinical importance. Some of the key structural findings that have been reported in children with ASD during early childhood include cortical surface hyperexpansion between 6 and 12 months of age in the middle occipital gyrus (bilaterally), right cuneus, right lingual gyrus, the left inferior temporal gyrus, and right middle frontal gyrus, followed by overall brain overgrowth between 12 and 24 months of age (Hazlett et al., 2017). Additional findings in this age range include age-specific differences in white matter tract development (Wolff et al., 2012), and distinct behavioral and cognitive developmental trajectories (Estes et al., 2015b).

Longitudinal studies using anatomical magnetic resonance imaging (MRI) can help to understand the structural changes that occur during early childhood (Hazlett et al., 2005, Hazlett et al., 2012b). In particular, morphometric techniques such as Voxel Based Morphometry (VBM) have been used to look at changes in the volume of white and grey matter in subjects with ASD (Chung et al., 2004, McAlonan et al., 2005). However, VBM may not be the best tool in the analysis of MRI data from early childhood, since it requires the accurate segmentation of grey matter (GM), white matter (WM) and cerebrospinal fluid (CSF)(Ashburner and Friston, 2000), a particularly difficult task due to the evolving myelination process occurring during the first year of life (Barkovich et al., 1988).

One alternative to VBM that does not rely on the accuracy of tissue segmentation is known as Tensor Based Morphometry (TBM). TBM is a technique that extracts information from the deformation fields resulting from a non-linear registration to an appropriate template and can be used to observe morphological changes in individual subjects in a longitudinal study, allowing the comparison of these changes between groups (Ashburner and Friston, 2004, Lau et al., 2008). Like VBM, one of the advantages of TBM is that there is no restriction to *a priori* regions of interest, but rather a voxel-by-voxel statistical analysis that yields the location of anatomical differences, including differences within a region, even if there are no evident full-brain or regional size differences (Ashburner and Friston, 2004, Boddaert et al., 2004, Chung et al., 2004). Longitudinal TBM studies have not been performed to analyze potential neurodevelopmental differences in early childhood (before 2 years of age) in ASD.

In the present study, a longitudinal analysis using TBM in the whole brain was performed on subjects between 6 and 24 months of age, including subjects at high familial risk (defined below) for ASD who later receive a diagnosis at 24 months, high-risk infants who do not receive a diagnosis at 24 months, and low-risk, typically developing controls. In order to tackle some of the complications of MRI studies in early childhood (e.g. reduced WM and GM contrast), we propose the use of both T1 and T2-weighted images simultaneously for the optimization of non-linear registration. Contrast changes due to myelination occur at different times in T1 and T2-weighted images (Barkovich et al., 1988), thus providing complimentary information for robust registration. Additionally, we incorporate the use of unbiased, age-appropriate templates as our registration targets. Our findings demonstrate significant differences in the growth trajectories of multiple regions in the brain between the high-risk ASD-positive group and both the high risk ASD-negative and low-risk controls, with some regions showing a faster growth rate and others showing a slower growth rate.

## 2 Methodology

### 2.1 Participants

All participants were part of the Infant Brain Imaging Study (IBIS), a multi-site collaborative, longitudinal study of infants at high and low risk of developing ASD based on their family history. It has been shown that siblings of children with ASD are at a higher risk of developing ASD themselves, with a reported risk as high as 18.7% (Ozonoff et al., 2011). Specifically, in this study infants were defined as *high risk* (HR) if they have an older sibling with a community-based, ASD diagnosis confirmed by the Social Communication Questionnaire (SCQ)(Rutter et al., 2003b) and the Autism Diagnostic Interview-Revised (ADI-R)(Rutter et al., 2003a). Infants were included in the *low risk ASD-negative* group (LR-) if they have at least one typically developing older sibling confirmed with the SCQ and no first-degree relatives with a developmental disability and did not meet clinical best estimate diagnosis of ASD at 24 months.

Recruitment, screening and assessment of the participants were performed at each of four sites: University of North Carolina, University of Washington, Children’s Hospital of Philadelphia, and Washington University in St. Louis. Participants were excluded from the study if they fulfilled any of the following general criteria: evidence of a specific genetic condition or syndrome, any significant medical condition potentially affecting neurodevelopment, significant vision or hearing impairments, low weight at birth (<2000 g), birth prior to 36 gestational weeks, significant perinatal adversity, pre-natal exposure to neurotoxins, any contraindication for MRI, predominant home language other than English, adopted children or half siblings, first-degree relative with psychosis, schizophrenia, bipolar disorder, or if they were twins.

All study procedures required the informed, written consent from the parents or legal guardians of all participants, as well as approval by institutional review at each site. All infants enrolled in the study were seen multiple times at 6, 12, and/or 24 months of age for MRI scanning and developmental and behavioural evaluation.

Using the complete diagnostic assessment at 24 months of age, the HR group was split into *high risk ASD-positive* (HR+) and *high risk ASD-negative* (HR-) subgroups. The HR+ group was defined by familial risk and diagnostic outcome based on the clinical best estimate made by experienced, licensed clinicians using the Diagnostic and Statistical Manual of Mental Disorders, 4th Edition, Text Revision (DSM-IV-TR) (American Psychiatric Association, 2000) checklist and supported by all available behavioural assessment data including the Autism Diagnostic Observation Schedule (ADOS) (Lord et al., 2000) and the ADI-R (Rutter et al., 2003a). The remaining HR subjects were included in the HR- group. For this study, we compared MRI scans from LR-, HR-, and HR+ infants that passed quality control. These criteria yielded 503 total participants (1,088 total scans) that included 162 LR- (346 scans), 285 HR- (627 scans) and 56 HR+ (115 scans). From these participants, 40% provided data for at least two time points, with 38.2% providing three time points. Demographic data for study participants as detailed in Table 1.

**Table 1:** Demographic Data.

	High-risk ASD-positive	High-risk ASD-negative	Low-risk ASD-negative	p-value
Total Participants	56	285	162	
6 m scans	40	202	137	
12 m scans	35	222	116	
24 m scans	40	203	93	
Participants with 1 time-point	18	53	39	
Participants with 2 time-points	17	122	62	
Participants with 3 time-points	21	110	61	
Age (6 m scan)^1^	6.6 (0.6)	6.7 (0.7)	6.8 (0.7)	0.71
Age (12 m scan)^1^	12.8 (0.5)	12.8 (0.7)	12.9 (0.8)	0.76
Age (24 m scan)^1^	25 (0.7)	25 (0.9)	25.1 (1.1)	0.74
ADOS severity^1^	6.1 (1.8)	1.5 (1.0)	1.3 (0.8)	<0.001
Sex (% male)^1^	85.6	58.3	59.3	<0.001

1 Omnibus ANOVA(Age, ADOS severity) and Chi-square test (Sex).

### 2.2 Image acquisition

The acquisition of all MRI scans was carried out at the four sites identified above during natural sleep, on identical 3 T Siemens TIM Trio scanners with 12-channel head coils using the following protocols: sagittal T1 MPRAGE (repetition time = 2400 ms, echo time = 3.16 ms, slice thickness = 1 mm, field of view = 256 mm, 256 × 160 matrix), 3D T2 fast spin echo (repetition time = 3200 ms, echo time = 499 ms, slice thickness = 1 mm, field of view = 256 mm, 256 × 160 matrix). Quality assurance was achieved using local Lego phantoms and travelling human phantoms over time to characterize intra- and inter-site reliability (Gouttard et al., 2008). Quality control for each scan included automatic verification of acquisition protocol parameters and visual assessment for potential artifacts due to subject motion, blood flow, or hardware issues.

### 2.3 Longitudinal tensor-based morphometry

#### 2.3.1 Preprocessing

All the scans were corrected for geometric distortion using data collected from the Lego and travelling human phantoms (Fonov et al., 2010). Intensity nonuniformity artifacts were corrected using the nonparametric nonuniform intensity normalization (N3) algorithm (Sled et al., 1998), followed by a histogram-based intensity normalization between 0.0 and 100.0, where the histogram is separated into deciles and the best linear mapping of the histograms is calculated.

#### 2.3.2 Age-appropriate average templates

T1 and T2-weighted unbiased average templates were created for each time-point in the longitudinal analysis (6, 12 and 24 months) according to the methods proposed by Fonov *et al.* (Fonov et al., 2011). This method consists of an intensity-matching algorithm that iteratively performs non-linear registration to first obtain an average T1-weighted template. The T2-weighted images were then warped using the deformation field obtained during the creation of the T1-weighted template and averaged together to create the T2-weighted template. This procedure ensures that both average templates are in the same space. All available scans from the IBIS database were used in the creation of the templates, including children from both the high risk and low risk cohorts. As such, each template is made from subjects that are approximately within 1 month of the mean population age. It is important to note that while there is a large contrast change over the period from 6 to 24 months, the contrast changes are much smaller between 5 and 6 months or between 12 and 13 months for example, making it possible to use these templates as anchors in our registration process.

#### 2.3.3 Registration

Each scan was linearly registered to the corresponding unbiased age-appropriate templates using Revised BestLinReg, a 5-stage hierarchical linear registration technique based on a normalized mutual information similarity measure (Dadar et al., 2018). This was followed by a non-linear registration using the symmetric image normalization (SyN) method (Avants et al., 2008), a symmetric diffeomorphic image registration algorithm. All non-linear registrations were obtained by simultaneously optimizing the cross-correlation between the T1 and T2-weighted scans and their corresponding unbiased templates (Table 2 shows the SyN registration parameters).

**Table 2:** SyN non-linear registration parameters.

Parameter	Value
Gradient Step	0.25
Update Field Variance in Voxel Space	2.0
Total Field Variance in Voxel Space	0.3
T1-image Cross-correlation weight	0.5
T2-image Cross-correlation weight	0.5
Shrink Factors (per step)	16/8/4/2/1
Smoothing Sigmas in mm (per step)	9.44/7.08/4.72/2.36/0

In order to obtain the inter-template transformations, the 6 and 24 months templates were linearly registered to the 12 months template, followed by a non-linear registration procedure using the SyN method (Avants et al., 2008) and optimizing for T1 and T2-weighted images simultaneously.

**Fig. 1:**
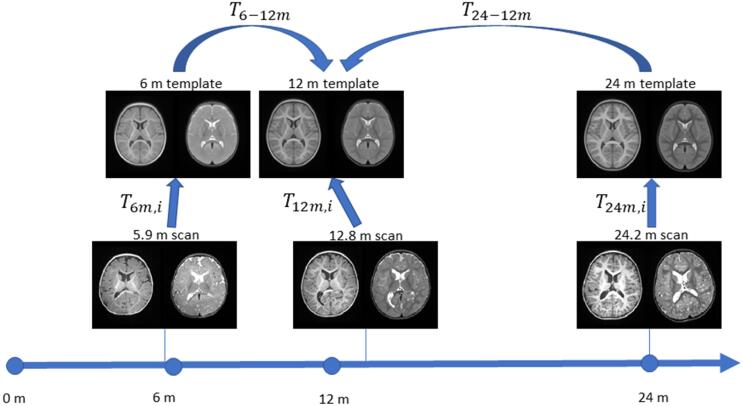
Visual representation of the registration process. Individual scans are registered to the age appropriate template using both T1 and T2 weighted images simultaneously. The 6 m and 24 m templates are registered to the 12 m template using a similar scheme. For an exemplary 5.9 months old subject the Jacobian determinant would be the result of: $\left|J\right|={{Concat(T}_{6m,i}T_{6-12m})}^{-1}$.

Finally, the non-linear deformation grids for each scan’s registration to an age-appropriate template were concatenated with the inter-template non-linear transformation, normalized and inverted, effectively yielding a voxel-by-voxel nonlinear mapping from the 12 months template reference space to the native space of each scan. A visual representation of this process can be seen in Fig. 1. By doing this, the individual differences of each subject to its age-appropriate template are conserved, while ensuring that all subjects at 6 and 24 months of age, respectively, undergo the same transformation from template to template. Furthermore, using only the non-linear deformation fields (i.e. excluding the linear transformations) in our method allows us to focus on local differences, as opposed to changes potentially driven by differences in overall brain size.

#### 2.3.4 Jacobian determinant

The natural logarithm of the Jacobian determinant of the deformation field was computed at every voxel and used as a surrogate of the local volume difference between each subject and the 12 m template. In general, a negative log-Jacobian determinant value represents shrinking from the template to the native space, a value of 0 indicates that there is no volume change in the voxel and a positive value indicates enlargement.

#### 2.3.5 Statistical analysis

Voxel-by-voxel tests of linear mixed-effects models were performed on the Jacobian determinant maps. These mixed-effects models are used to characterize the local growth trajectory of each voxel. Mixed-effects models are commonly used in longitudinal data analysis since they can deal with missing data while accounting for heterogeneity from different individuals by introducing subject-specific random effects (Cheng et al., 2010).

Forward model selection was used, beginning with the testing of the simplest growth trajectory model and subsequently adding variables that show statistical significance. The fixed effects that were evaluated included the linear, quadratic and cubic age effects (age, age^2^, age^3^), acquisition site, the effect of sex and group (LR-, HR-, or HR+), as well as any interactions between them (e.g., age*sex). The age in days of each participant was used in the model. For the random effects, both a random intercept and random slope were tested to account for within-subject dependencies, as well as a random intercept to account for potential inter-site differences. The mixed-effects models were compared using voxel-wise log-likelihood ratio tests of nested models. The more complex model was chosen when the log-likelihood ratio test showed a significantly better fit for the majority of the voxels and the mean F-statistic for all voxels was found significant after multiple comparisons correction. The selected model was applied to all voxels.

The Jacobian determinant maps provide a voxel-wise measure of local relative volume with respect to the 12-month reference space (Ashburner and Friston, 2004). After performing the mixed-effects models testing using the previously mentioned effects, the final statistical model tested in the whole brain included fixed-effects for age ($\beta_{1}$), age^2^ ($\beta_{2}$), group ($\beta_{3}$), the interaction between age and group ($\beta_{4}$), and sex ($\beta_{5}$), as well as a random effect for the intercept for each subject ($\gamma_{0i}$) and for acquisition site ($\gamma_{0site}$). Therefore, the model evaluated for each subject *i* at each voxel was:

(1) $$J_{i}\left(x\right)=\gamma_{0i}{+\gamma_{0site}+\beta }_{0}+\beta_{1}*age+\beta_{2}*age^{2}+\beta_{3}*group+\beta_{4}\left(group\times age\right)+\beta_{5}*sex+E$$

where $\beta_{0}$ represents the intercept and *E* is the residual error in the model.

The false discovery rate (FDR) procedure described by Genovese *et al.* (Genovese et al., 2002) was used to control for multiple comparisons with an FDR of 5%. A single t-value threshold was determined for each resulting statistical map by taking into account the estimated degrees of freedom for a given statistical test and an FDR p-value obtained by pooling the uncorrected p-values across all effects and all voxels tested (Lau et al., 2008). All statistical analyses were performed using the R software package (www.r-project.org) in conjunction with the *lme4* (Bates et al., 2015) and *RMINC* (Lerch et al., 2017) libraries.

## 3 Results

The results of the analysis identified voxels with significant changes in local volume that can be described by the parameters in the model. As such, a statistical map was obtained for each effect in the model, where each statistically significant voxel provides information about the local growth trajectory when the other effects are removed. Of particular interest to this study are the voxels found to be statistically significant for the interaction between age and group, since it signifies a region that is affected differently by age in each group and may be thus associated with a group-specific growth trajectory, indicating an increase or decrease in growth rate in the HR+ group compared to the HR- and LR- groups. The effect of group was not significant at any level at the centered age (6 months).

**Fig. 2:**
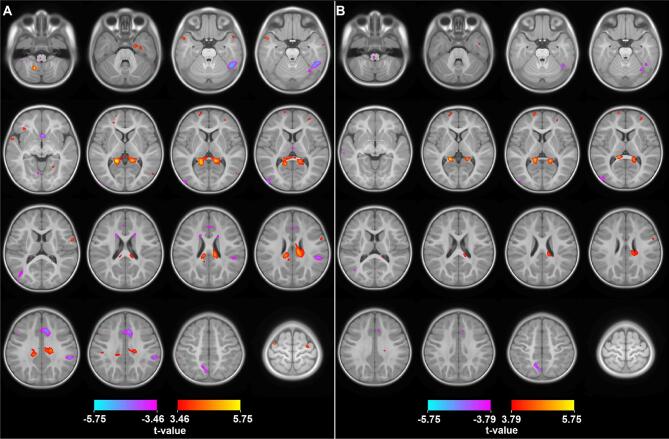
Statistical maps showing regions with significant differences in growth rate given by the age and group interaction. A) HR+ vs LR-; B) HR+ vs HR-. All colored regions are statistically significant for pooled FDR (q = 0.05). Since the number of subjects compared in A and B are different, the t-value thresholds are different to achieve q = 0.05 (see color bars). Note that the fitted growth curves for some of the peaks shown here are presented in Fig. 3, Fig. 4. Images follow neurological convention.

We used the Neuromorphometics atlas (Neuromorphometrics Inc., 2013) to identify the anatomical regions of groups of voxels showing significantly different growth trajectories. These anatomical regions, as well as the tissue type where they are located are summarized in Table 3. Representative slices showing the pairwise HR+ vs LR- and HR+ vs HR- comparisons with thresholded t-values (FDR of 5%) for the age by group interaction are shown in Fig. 2. As compared to the LR-, the largest connected region of increased growth rate associated with the HR+ group includes WM radiating from the splenium of the corpus callosum and the posterior cingulate gyrus bilaterally. Additional areas of increased growth rate in the HR+ group when compared to the LR- group include the cingulum bilaterally, the right parahippocampal gyrus and entorhinal area, and the left temporal pole and cerebellum. Significant regions showing decreased growth rate associated with the HR+ group compared to the LR- group include the anterior portion of the caudate bilaterally, the left precuneus, middle occipital gyrus and lingual gyrus, and the right fusiform gyrus, supplementary motor cortex, supramarginal gyrus and subgenual anterior cingulate cortex. Of particular note are regions with significantly different growth trajectories of the HR+ group when compared to both the HR- and LR- group, representing disorder specific effects as opposed to those related to genetic liability. These regions include increased growth rate in the posterior cingulate gyrus, WM radiating from the splenium, and the precentral gyrus bilaterally, as well as the right cingulum, and decreased growth rate in the right fusiform gyrus and inferior temporal gyrus, as well as the left precuneus.

**Table 3:** Regions with Significant Interactions between Age and Group.

Region	Cluster Size	Peak t-value		Tissue Type
		HR + vs LR-	HR + vs HR-	
Bilateral Posterior Cingulate Gyrus Splenium and Isthmus of the Corpus Callosum Bilateral White Matter	2131	5.74***	5.54***	Mixed
Right Cingulum Right White Matter	1587	4.95***	4.45**	WM
Right Fusiform Gyrus Right Inferior Temporal Gyrus	1242	−5.24***	−4.63**	GM
Right Supplementary Motor Cortex Right Superior Frontal Gyrus (Medial Segment)	964	−4.94***	−4.04**	GM
Left Cingulum Left White Matter	788	4.60***	3.56*	WM
Right Supramarginal Gyrus Right Parietal Operculum	773	−4.71***	−2.85	GM
Left Precuneus	388	−4.45***	−5.02***	GM
Left Middle Occipital Gyrus	312	−4.32***	−4.18**	GM
Left Cerebellum	302	5.37***	3.09	WM
Right Parahippocampal Gyrus Right Entorhinal Area	244	4.32***	2.98	GM
Subgenual Anterior Cingulate Cortex Right Accumbens	244	−5.02***	−3.68*	GM
Left Temporal Pole Left Superior Temporal Gyrus	233	4.93***	3.33*	GM
Left Posterior Orbital Gyrus Left White Matter	221	4.66***	3.42*	WM
Right Precentral Gyrus	173	4.24**	3.82**	GM
Right Occipital Fusiform Gyrus	151	−4.45***	−4.98***	GM
Left Temporal Pole	147	4.66***	3.16	GM
Left Caudate	146	−4.31***	−3.57*	GM
Right Caudate	117	−4.14**	−3.00	Mixed
Right Precentral Gyrus	115	4.17**	3.62*	GM
Right Fusiform Gyrus	95	4.21**	3.05	GM
Left Lingual Gyrus	72	−3.99**	−3.52*	GM
Left Precentral Gyrus	68	4.53***	3.91**	GM
Left Parietal White Matter	67	3.91**	3.48*	WM
Right Precentral Gyrus	60	4.00**	4.28**	Mixed
Left Frontal Pole	55	3.76**	4.45**	GM
Right Medial Orbital Gyrus	54	3.89**	1.74	GM
Right Middle Frontal Gyrus	50	3.89**	4.25**	GM
Right Superior Frontal Gyrus (Medial Segment)	45	−3.82**	−3.83**	GM
Right Middle Temporal Gyrus	44	3.94**	3.13	GM
Right Cerebellum	43	3.89**	4.11**	Mixed
Right Fusiform Gyrus	40	3.88**	4.07**	GM
Right Temporal Pole	39	3.77**	1.98	GM
Left Lateral Orbital Gyrus	35	−3.68**	−4.25**	GM

*Significant with FDR = 0.10; **Significant with FDR = 0.05, ***Significant with FDR = 0.01

**Fig. 3:**
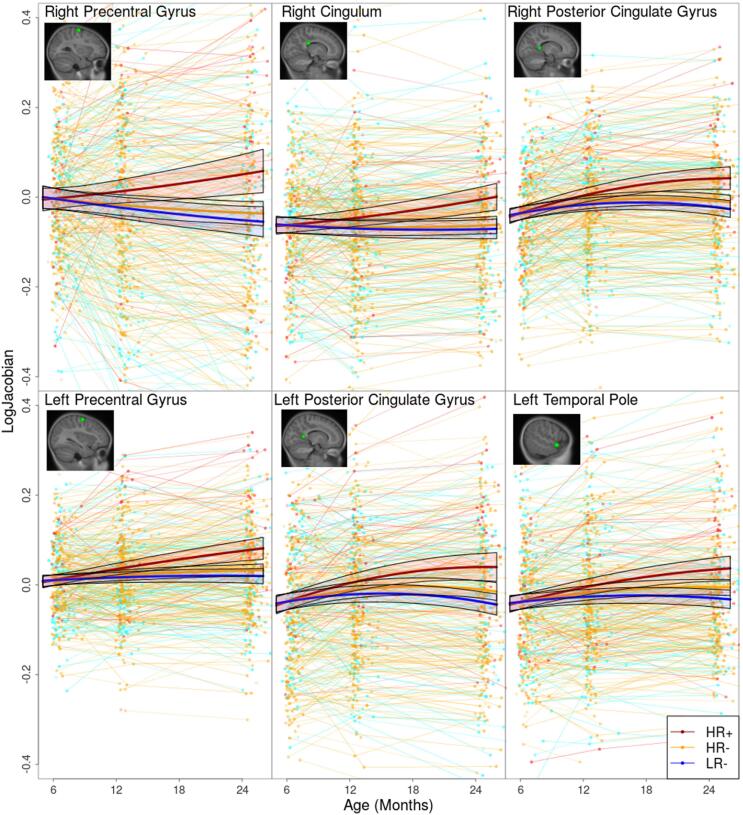
Voxelwise growth trajectories with 95% confidence intervals for selected significant regions showing increased growth rate in the HR+ group when compared to the HR- and LR- groups. Voxels were selected by looking at the peak t-values of both the HR+ vs HR- and HR+ vs LR- comparisons.

**Fig. 4:**
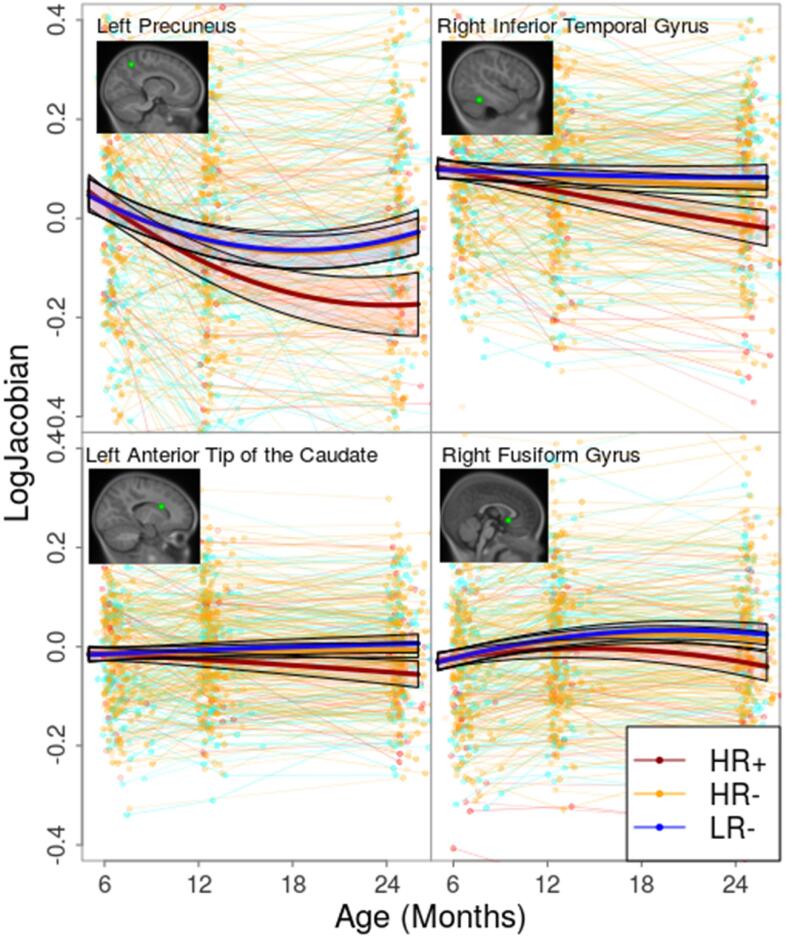
Voxelwise growth trajectories with 95% confidence intervals for selected significant regions showing decreased growth rate in the HR+ group when compared to the HR- and LR- groups. Voxels were selected by looking at the peak t-values of both the HR+ vs HR- and HR+ vs LR- comparisons.

To evaluate the magnitude and time-course of these differences, Fig. 3 shows exemplar voxel-wise growth trajectories demonstrating distinct increased local growth rate in the HR+ group in 6 different regions distributed across the brain. These regions include the left temporal pole, right posterior cingulate gyrus, left posterior cingulate gyrus, left precentral gyrus, right precentral gyrus and the right cingulum. Additionally, Fig. 4 shows trajectories with decreased local growth rate in the HR+ group in four different regions. These regions include the right fusiform gyrus, left anterior tip of the caudate, left precuneus and the right subgenual anterior cingulate cortex.

## 4 Discussion

In this longitudinal study, we found a pattern of significant differences in growth in the HR+ group when compared with the HR- group and the LR- controls (see Fig. 2). Several regions across the brain show different growth patterns in the HR+ group, either as an increased (Fig. 3) or a decreased (Fig. 4) growth rate. In general, the regions found to have decreased growth rate are found in GM, while regions with increased growth rate are mostly found in WM. This inverse behaviour in ASD could be tied to the growth patterns in normal neurodevelopment, where GM experiences very rapid growth during the first 2 years of life, while WM undergoes a slower, steady growth (Knickmeyer et al., 2008).Overall, regions found to have increased growth outnumber the regions with decreased growth, this effect could be tied to the well documented general brain overgrowth in children with ASD. Furthermore, the predominance of increased growth rate in WM is consistent with previous studies showing volume increases across WM in ASD (Hazlett et al., 2011, Herbert et al., 2004). Additionally, the differences between groups found here are dependent on age, with the growth trajectories diverging with increasing age (see Fig. 3).

Our results are in agreement with previous studies of ASD within the same age range, showing no significant volumetric differences at 6 months of age (Hazlett et al., 2012a), but rather distinct growth trajectories leading to overgrowth that begin to diverge later in life, between 12 and 24 months of age, as shown in total brain volume changes by Hazlett et al. (Hazlett et al., 2017). In this work, Hazlett et al. developed a deep-learning algorithm using cross-sectional features based on surface area information of 6 and 12-month old individuals to predict the diagnosis of ASD in individual high-risk children at 24 months with a positive predictive value of 81% and a sensitivity of 88%. Interestingly, 40% of the anatomical regions used in their deep learning framework show significant differences in the longitudinal growth trajectories estimated with the data-driven TBM methodology used here. Some of the more important trajectory differences include the medial portion of the right superior frontal gyrus, the left lingual gyrus, and the left precuneus.

In the case of the regions with increased growth found in the cerebrum, the splenium of the corpus callosum has been associated with language production in typically developing children between 6 and 24 months of age (Swanson et al., 2015), and therefore differences in this region might be associated with deficits in communication, one of the core symptoms in ASD. This region has also been implicated in visual orienting abilities in typically developing 7-month-olds, an association not observed in infants later showing signs of ASD (Elison et al., 2013). Findings in the corpus callosum are of particular importance when considering that the axons that form the corpus callosum are predominantly involved in long-distance connections, with previous research showing long-range functional and anatomical underconnectivity in adults with ASD (Horwitz et al., 1988, Just et al., 2012, Kana et al., 2009) and overconnectivity in children with ASD (Supekar et al., 2013). The posterior cingulate gyrus has been previously implicated in social impairments observed in ASD, particularly the self and other reflection (Chiu et al., 2008, Kennedy and Courchesne, 2008). The cingulum bundle has an important role in the connectivity required for social cognition (Amodio and Frith, 2006) as well as emotional processing (Bush et al., 2000), and has been previously reported to show differences in WM integrity in ASD as early as 2 to 3 years of age (Weinstein et al., 2011, Xiao et al., 2014). Motor impairments present in ASD have been previously associated with differences in the precentral gyrus, particularly as increases in WM volume in children between 8 and 12 years of age (Mostofsky et al., 2007).

The regions showing a decrease in growth rate are found mainly in GM and are in general smaller in magnitude and size than those showing an increased growth rate. The biggest cluster with decreased growth rate in ASD is found in the right fusiform gyrus and includes a part of the right inferior temporal gyrus. The right fusiform gyrus has been consistently reported to be involved in face processing tasks (Allison et al., 1994, Puce et al., 1996), and has been found to have increased volume in adolescents and adults with ASD (Rojas et al., 2006, Waiter et al., 2004). Alterations in the right fusiform gyrus have been shown to vary with age in adolescents and adults (Raznahan et al., 2010, Wallace et al., 2010), as such, the decreased growth rate at this early age might be tied to future normalization and overgrowth in later stages. A similar situation occurs with the right inferior temporal gyrus and the left precuneus, where increased volumes have been associated with ASD in adolescents and adults (Liu et al., 2017). The left precuneus and the left lingual gyrus have also been found to have decreased cortical thickness in older subjects with ASD (Pereira et al., 2018). Little is known about potential alterations during early childhood, thus highlighting the importance of looking for longitudinal changes in neurodevelopmental patterns across the different age ranges. In addition, we found a decreased growth rate in the subgenual anterior cingulate cortex. This region is implicated in the inhibition of the amygdala and emotion regulation (McDonald, 1998, Ray and Zald, 2012, Stevens et al., 2011) and is known to play an important role in various mood disorders (Drevets et al., 2008). Furthermore, it has been recently associated with ASD in rat models (Wu et al., 2018) and in older children and adolescents (Velasquez et al., 2017).

We found two potential conflicts of the present results with previous studies. First, a study by Wolff *et al*. (Wolff et al., 2015) examined the length, area and thickness of the corpus callosum in subjects from the IBIS database in the same age range (6–24 months). They reported a significantly greater area and thickness of the normalized corpus callosum in the HR+ subjects at six months, decreasing to a non-significant difference at 24 months. Our results show an increased growth rate in the splenium throughout the age range. There are several potential explanations for this apparent discrepancy. First, the metrics analyzed in both studies are different, the shape analysis described in Wolff *et al*. uses explicit shapes and measures thickness, length, and area. Our voxel-based deformations measure local changes which do not really capture “object-level” differences. Second, the normalization procedure is different. In Wolff *et al.*, corpus callosum metrics were normalized for brain volume, sex, site, mother’s education and Mullen Early Learning. The TBM method here registers all data to an average 12-month template, and the Jacobian determinant is used to estimate voxel-by-voxel growth on a per-subject basis. The Jacobian values are thus normalized to the size of the average 12-month old brain. Brain volume is accounted for in the TBM registration process, while sex and site are included in our mixed-effects model. The choice of a normalization strategy is well known to have an important impact on results, particularly for the corpus callosum (Smith, 2005). Additionally, Wolff *et al.* looked at group differences in the size of the corpus callosum at 6, 12, and 24 months, while the present study focuses on differences in the growth trajectories, particularly the growth rate as affected by the group and age interaction. Finally, both the results of Wolff *et al.* and our results show enlargement of the corpus callosum in ASD at an early age, in contrast with studies reporting smaller corpus callosum in older children and adults (Frazier et al., 2012, Hardan et al., 2009, 2000), highlighting a continuing change over time of the brain in ASD.

Additionally, we found small regions at the tip of the anterior caudate nuclei to have a decreased growth rate, with no significant difference in the body of the caudate nuclei. The caudate nuclei has been reported to have an increased growth rate and larger overall volumes in older children with ASD (Langen et al., 2014, Qiu et al., 2016). One potential explanation for this discrepancy is that, as the caudate begins to enlarge, the small section adjacent to the ventricular horn, or the ventricular horn itself, are slightly compressed, causing our method to detect a decreased growth rate in a very small portion of the caudate, while in reality the pattern of overgrowth in the caudate volume is increasing but not yet significant.

The limitations of the present study are partially seen by the previously mentioned conflicts. Due to the reliance of TBM on the non-linear registrations, small regions (e.g. the corpus callosum and the anterior tip of the caudate) can be affected by changes in the opposite direction in its neighbouring structures. Furthermore, the contrast between GM and WM in the images changes with age. These changes in contrast affect the quality of the non-linear registration, especially in the data acquired at around 6 months of age, where brain regions undergo contrast reversal, leaving no visible boundary between GM and WM. The registration algorithm might then simply interpolate these regions, with the Jacobian being the result of the choice and scale of interpolation rather than image texture changes. Our methodology is designed to mitigate this problem, mainly with the simultaneous use of T1 and T2-weighted images for registration and by choosing a relatively large gradient step and small field variance penalties in order to avoid overly smooth fields and favor local high-resolution changes. The WM and GM contrast due to myelination changes differently in T1 and T2-weighted images and, by leveraging this time-shift between modalities, we provide additional information resulting in more accurate registrations at an early age. However, the myelination process and its effects on T1 and T2-weighted contrast are complex, and some regions may still have no clear boundaries or sufficient information for accurate registration.

In conclusion, these results detail, at voxel level, growth differences previously documented as increased brain volume. These voxel level measures of growth differences indicate that the local ASD-associated patterns of growth are more complex than has been inferred from the global patterns, with regions showing growth differences in either direction. Further, many of these regions are involved in social information processing, emotion and language, all of which are known to be impaired in ASD. This information, if corroborated with brain-behavior associations may inform the search for more specific, targeted interventions.

## CRediT authorship contribution statement

**A. Cárdenas-de-la-Parra:** Conceptualization, Methodology, Software, Validation, Formal analysis, Visualization, Writing - original draft. **J.D. Lewis:** Conceptualization, Writing - review & editing. **V.S. Fonov:** Data curation, Software, Methodology. **K.N. Botteron:** Writing - review & editing. **R.C. McKinstry:** Writing - review & editing. **G. Gerig:** Writing - review & editing. **J.R. Pruett:** Writing - review & editing. **S.R. Dager:** Writing - review & editing. **J.T. Elison:** Writing - review & editing. **M.A. Styner:** Writing - review & editing. **A.C. Evans:** Writing - review & editing. **J. Piven:** Writing - review & editing, Project administration, Funding acquisition. **D.L Collins:** Conceptualization, Supervision, Writing - review & editing.

## Declaration of Competing Interest

The authors declare that they have no known competing financial interests or personal relationships that could have appeared to influence the work reported in this paper.
